# Direct and indirect effects of different types of microplastics on freshwater prey (*Corbicula fluminea*) and their predator (*Acipenser transmontanus*)

**DOI:** 10.1371/journal.pone.0187664

**Published:** 2017-11-06

**Authors:** Chelsea M. Rochman, J. Mark Parnis, Mark A. Browne, Sebastian Serrato, Eric J. Reiner, Matthew Robson, Thomas Young, Miriam L. Diamond, Swee J. Teh

**Affiliations:** 1 Department of Ecology and Evolutionary Biology, University of Toronto, Toronto, Ontario, Canada; 2 Aquatic Health Program, School of Veterinary Medicine, University of California, Davis, Davis, California, United States of America; 3 Chemical Properties Research Group, Department of Chemistry, Trent University, Peterborough, Ontario, Canada; 4 Evolution & Ecology Research Centre, School of Biological, Earth & Environmental Sciences, University of New South Wales, Sydney, NSW, Australia; 5 Ontario Ministry of the Environment and Climate Change, Toronto, ON, Canada; 6 Department of Civil and Environmental Engineering, University of California, One Shields Ave., Davis, CA, United States of America; 7 Department of Earth Sciences, University of Toronto, Toronto, Ontario, Canada; VIT University, INDIA

## Abstract

We examined whether environmentally relevant concentrations of different types of microplastics, with or without PCBs, directly affect freshwater prey and indirectly affect their predators. Asian clams (*Corbicula fluminea*) were exposed to environmentally relevant concentrations of polyethylene terephthalate (PET), polyethylene, polyvinylchloride (PVC) or polystyrene with and without polychlorinated biphenyls (PCBs) for 28 days. Their predators, white sturgeon (*Acipenser transmontanus*), were exposed to clams from each treatment for 28 days. In both species, we examined bioaccumulation of PCBs and effects (i.e., immunohistochemistry, histology, behavior, condition, mortality) across several levels of biological organization. PCBs were not detected in prey or predator, and thus differences in bioaccumulation of PCBs among polymers and biomagnification in predators could not be measured. One of the main objectives of this study was to test the hypothesis that bioaccumulation of PCBs would differ among polymer types. Because we could not answer this question experimentally, a bioaccumulation model was run and predicted that concentrations of PCBs in clams exposed to polyethylene and polystyrene would be greater than PET and PVC. Observed effects, although subtle, seemed to be due to microplastics rather than PCBs alone. For example, histopathology showed tubular dilation in clams exposed to microplastics with PCBs, with only mild effects in clams exposed to PCBs alone.

## Introduction

Diverse animals are exposed to microplastics and associated chemical contaminants due to their ubiquity in aquatic environments globally [1, 2]. Research on microplastics in freshwater is increasing. Initial research shows the widespread occurrence of microplastics in lakes [3–5] and rivers [6–8]. These findings raise concern about the impacts of microplastics on freshwater biota [9]. The aim of this study was to improve scientific understanding about impacts from microplastics to freshwater organisms by measuring direct and indirect effects to a model freshwater prey and their predator. This study also aimed to measure impacts to freshwater biota from environmentally relevant concentrations of microplastics and to explore how toxicological effects and bioaccumulation of chemicals differ among polymers.

Laboratory studies have investigated effects from microplastics in a range of animals (e.g., lugworms [10], oysters [11], crabs [12] and fish [13]) and across several levels of organization, most of which are suborganismal and some that are more ecologically relevant (e.g., levels that affect populations, assemblages; [14]). Still, further studies examining effects are necessary, and particularly studies that are more environmentally relevant [14]. Environmentally relevant studies include those that investigate animals likely to be contaminated in nature and that use concentrations and exposure conditions that are similar to what is found in nature (e.g., plastic types, shapes and sizes, and realistic mechanisms of exposure and exposure durations). Moreover, only a few studies have measured effects on freshwater organisms [15–18]. Most studies thus far still have focused on marine animals. Finally, few studies have compared impacts across polymers, a consideration that is important for policy and industry, since there may be effects from the particles themselves [19] and/or from the chemicals unique to different polymers [20].

In this study, experiments used four common microplastic types: polyethylene terephthalate (PET), polyethylene, polyvinyl chloride (PVC) and polystyrene. Polymers were chosen based on their different capacities to accumulate priority pollutants and their propensity for release of constituent monomers that are considered hazardous. Polyethylene and polystyrene sorb significantly greater concentrations of priority pollutants than PET and PVC [21]; but, PVC and polystyrene contain the hazardous constituent monomers vinyl chloride and styrene, respectively [20]. These four polymers also comprise more than half of the plastic produced [22] and that are found as litter in marine and freshwater habitats [23].

Polychlorinated biphenyls (PCBs) were used as model priority pollutants because they are widespread, continue to be released into the environment [24], are a threat to wildlife [25], and are found on microplastics globally [26]. A mixture of isotopically labeled coplanar PCBs, which have dioxin-like properties and toxicity (^13^C labeled PCB# 77, 81, 126 and 169; [27, 28]) were selected. Isotopic labels were used to allow measurement of transfer and bioaccumulation of PCBs at low concentrations without confounding due to background concentrations.

The model species used are ecologically relevant and commercially important—the Asian clam, *Corbicula fluminea* and its predator white sturgeon, *Acipenser transmontanus* [29]. *C*. *fluminea* is found throughout freshwater bodies and is now one of the most common freshwater bivalves in North America [30]. While they are a favorable food source in Korea [30], consumption by humans in North America is discouraged due to the ability of the clams to accumulate large concentrations of pollutants. As a filter feeder, the Asian clam has the potential to contain large amounts of microplastics. In Taihu Lake in China, Asian clams contained 0.2–12.5 microplastic particles/g ww [31]. Many species of sturgeon are threatened as a consequence of habitat-degradation [32] and commercial harvesting, especially for eggs [33].

The objective of this study was to measure three potential mechanisms by which different types of microplastics with or without PCBs could directly affect a freshwater prey species and indirectly affect their predator, due to 1) different polymers that vary in their occurrence of hazardous monomers, 2) the presence of PCBs, and 3) the interaction between microplastics and PCBs. We hypothesized that bioaccumulation and toxic effects would vary across polymers and among species. Bioaccumulation of PCBs was measured in clams and fish and modelled in clams to examine differences in bioaccumulation among polymers and to assess trophic transfer. To measure direct effects to prey and indirect effects to their predators, we chose effects that have been tested and demonstrated in our previous studies [10, 15, 34] and that are relevant to several levels of biological organization. These include changes in the expression of proteins relevant to metabolism of toxicants (CYP450) and endocrine system function (vitellogenin), changes in tissues via histopathology, changes in feeding behavior and mortality in both prey and predator.

## Materials and methods

### Experimental design

Experiments included 10 treatments: negative control (no plastic and no PCBs), positive control (no plastic with PCBs), PET, polyethylene, PVC, polystyrene, PET+PCB, polyethylene+PCB, PVC+PCB and polystyrene+PCB. Treatments were designed to measure effects related to different polymers, PCBs and the combination of microplastics with PCBs. Due to our large diversity of treatments, and thus need for many animals and tanks, our experiments have a small sample size (n = 3 tanks per treatment).

### Animal collection and permitting

Permits from the U.S. Department of Fish and Wildlife (Permit ID# 12783) allowed us to collect *C*. *fluminea*, ranging in size from 16–30 mm and averaging 20.8 mm ± 2.4 Std. Dev., from Putah Creek in Davis, California, USA. Clams were depurated for two days in clean water prior to acclimation in their experimental tanks. Sturgeon (2 days post-hatch; from Lazy Q Farms, Dixon, CA) were raised in our laboratory until they weighed more than five grams (~45 days). This study was approved by the Institutional Animal Care and Use Committee (IACUC), University of California-Davis and followed the experimental protocol for Animal Care and Use #17846.

### Preparation of microplastics

Animals are not exposed to uniform sizes of microplastics in nature. They are exposed to a variety of shapes and sizes. To account for this, pre-production plastic pellets of different types were micronized using an industrial grinder to make separate mixtures of microplastics, each with a similar size range and fragmented shape. Pre-production pellets of PET, polyethylene, PVC and polystyrene (SABIC Innovative Plastics, Mt Vernon, IN, USA) were micronized by Custom Processing Services (Reading, PA, USA). The size distributions and mean of each type of microplastic were similar, but not perfectly uniform due to differences in how the polymers micronized. PET ranged in size from 12 to 704 μm (mean 198 μm), polyethylene from 14 to 704 μm (mean 209 μm), PVC 80–704 μm (mean 169 μm) and polystyrene 68 to 704 μm (mean 179 μm). These size ranges fall within the range of those reported in nature [35]. Although these sizes seem bigger than what would be expected for a small bivalve to eat, reports in the literature substantiate that small bivalves ingest microplastics in the size range used here [36, 37], including Asian clams [31]. Still, to assure the size range was appropriate for our study, we conducted a pilot study that confirmed that clams in this study can and will ingest the plastics.

Prior to exposures, a pilot study (3-day) confirmed that clams would ingest each type of microplastic. Using one tank per treatment (including a no-plastic control) and 5 clams per tank, we exposed clams to each type of microplastic using the same size, shape and concentrations of microplastics as in the experiments. After the exposure, clams were digested in 10% potassium hydroxide (KOH; [38]). Results showed that microplastics were present in all 5 clams individually from all treatments and controls had no plastic. Individual clams had an average of 5 ± 6 particles of PET, 8 ± 6 particles of polyethylene, 3 ± 3 particles of PVC, and 4 ± 3 particles of polystyrene.

To prepare treatments of each polymer and one of algae with PCBs for the dietary exposures, each type of microplastic (100 grams each in 500 mL milliQ water) and algae (1 L *Pavlova* sp., 4–7 μm, from Reed Mariculture, Campbell, CA, USA) were placed into separate clean (baked at 450°C for 6 hours) amber glass bottles and spiked with coplanar PCBs to achieve 30 ng/g (^13^C-labeled PCB#77, 81, 126 and 169 in Nonane; Wellington Laboratories, Guelph, Canada). This concentration was based on what was found on microplastics in the San Diego Bay, CA, USA after being deployed in Bay water for one year [21]. This concentration is at the lower end of the range of concentrations found on microplastics in nature [26]. The amount of PCBs that was added to each glass jar with microplastics or algae was calculated assuming sorption would follow a linear adsorption isotherm and using partition coefficients gathered from the literature for each polymer [39, 40, 21] and algae [41]. Amber glass bottles containing water, PCBs and microplastic were tumbled for a 2-week period at room temperature. Following the 2-week period, microplastics were filtered from water using clean glass fiber filters, dried, stored in clean glass jars and kept at -20°C until experimentation and chemical analysis. To maintain the integrity of the algal cells, algae spiked with PCBs were maintained in a water bath (25°C) and gently mixed each day for 4 days. Following this, the algae and water mixture was maintained in a glass jar at -20°C until experimentation and analysis.

### Dietary exposures

*C*. *fluminea* were randomly placed into tanks within one of two experimental systems. The main experimental system held 30 individual 4 L tanks (3 tanks per treatment with 30 clams each). The other system, maintained to prepare clams to be fed to sturgeon in the following experiment, held 10 individual 50 L tanks (1 tank per treatment with 350 clams each). Both systems were semi-static, kept on a 16-hour light-cycle and maintained at 22°C using a recirculating water bath. Water quality parameters, including pH (7.8 ± 0.2), ammonium and nitrite (not detectable), nitrate (<0.5 ppm), hardness (100–120 mg L^-1^ CaCO_3_), electrical conductivity (280 UMHOS) and alkalinity (80 mg/L CaCO_3_), were maintained and monitored by testing water in three random individual tanks per week. During acclimation and experimentation, clams were fed brown algae (*Pavlova* sp., as described above) three days/week. Clams in the small tanks (main experimental system) were fed 0.8 g of algae and clams in the large tanks (other system of larger tanks for preparation of diet for sturgeon) were fed 8.8 g of algae. The walls of each tank were cleaned every other day. Clams acclimated to the experimental system for 2 weeks prior to experimentation.

During the 28-day exposure period, clams were exposed to concentrations of microplastic that equaled 0.0003% by volume of water. Because densities vary by polymer type, this equates to exposure concentrations of 4.1 mg/L for PET, 2.8 mg/L for polyethylene, 4.2 mg/L for PVC and 3.2 mg/L for polystyrene. These concentrations are comparable to higher concentrations of microplastics that have been recorded in freshwater environments (up to 100 particles per L; [9]). Although this does not provide a mass per L, concentrations are more than three orders of magnitude greater than maximum concentrations measured in the N. Pacific Gyre (0.03 particles per L, which equated to 0.25 mg/L; [42]). Concentrations in our study are one order of magnitude greater than in the N. Pacific Gyre and thus likely relevant to mass concentrations reported in freshwater environments.

Each day, 100% of the water in each small tank was exchanged to avoid accumulation of microplastics in the tanks. In the large tanks, only 60% of the water was exchanged daily to conserve water. Because we did not do 100% water changes in the large tanks, we were careful to siphon from the bottom, surface, and from the water column. Still, we recognize that the concentration of plastics in this system was slightly less controlled than in the small tanks. After water exchanges, clams in each tank were dosed with 0.0003% plastic (or PCB-spiked algae for the positive control) by volume of water in the tank per day. The volume of each polymer or PCB-spiked algae added to tanks in each treatment was obtained as the product of mass and polymer density. Clams were exposed to microplastics by sprinkling it at the top of their tank. Each tank was equipped with an air bar positioned parallel across the bottom which kept the water and all microplastics circulating in the tank during experimentation. We did not see particles sinking to the bottom, but did not count the microplastic particles in the water column during experimentation. Thus, we cannot guarantee that some particles did not sink to the bottom. Still, the availability of particles for ingestion should be similar as Asian clams feed via both filter- and deposit- feeding. Mortality was recorded daily.

After 28-days, clams from the big tanks were sampled and stored at -20°C to prepare diets for the sturgeon. In the small tanks, feeding behavior was measured on day 29 (see details below) and all clams were sampled on day 30 following a 48-hour depuration period for microplastics. All clams from the small tanks were stored for future analysis of several parameters including bioaccumulation of PCBs, histopathology and immunohistochemistry.

Juvenile sturgeon were exposed to the same 10 treatments of clams as described above. Sturgeon diets were prepared using a mixture of 126.4 g vitamin free casein, 61.1 g wheat gluten, 111.1 g dextrin, 16.3 g egg albumin, 21.4 g soy lecithin, 8.1 g vitamin premix, 12.2 g mineral premix, 8.2 g corn oil, 20.4 g cod liver oil, 8.0 g celufil and 100 g wet weight of homogenized clams from the large tanks in the first experiment (diets were 20% clams by weight). Vitamin and mineral mixes were purchased from ICN Biomedical, Inc. (Irvine, CA) and all other ingredients from U.S. Biochemical Corporation (Cleveland, OH).

Sturgeon (average mass 11.7 ± 4 g and length 145.2 ± 17.5 mm) were randomly divided into thirty 60 L tanks (three replicate tanks per treatment and 12 fish per tank). The flow-through system was maintained at a constant temperature (19 ± 1°C) on a 16-hour light-cycle. Water quality parameters were monitored weekly and maintained as follows: pH (8.0 ± 0.2), ammonium and nitrite (not detectable), nitrate (<3 ppm), water hardness (400–420 mg L^-1^ CaCO_3_), electrical conductivity (740 UMHOS) and alkalinity (400 mEq/L).

Fish were acclimated to their experimental tanks for 7 days before commencing the 28-day dietary exposure. Twice a day, fish were fed 1.5% of their body weight, split into two equal portions. Fish were weighed weekly and the amount of diet altered according to their weight. Before diets were sprinkled at the top of each tank, the flow of water to all tanks was shut off for 30 minutes to reduce the loss of diet and to allow time for feeding. Mortality was recorded daily. On day 29 we conducted a feeding behavior study (see details below). On day 30, after a 48-hour depuration period, fish were sampled for the same suite of endpoints as the clams. Length and weight were measured to assess condition factor. At this time, fish were still too young to determine their sex.

### Chemical analysis of PCBs

All solvents were pesticide or high-performance liquid chromatography (HPLC) grade (Fisher Scientific, Fair Lawn, NJ, USA). ^13^C labeled PCB#81, 77, 126 and 169, used as target compounds, and ^13^C PCB#189, used as a recovery standard, were purchased from Wellington Laboratories (Guelph, Canada). PCB#65, 155 and 204, used as internal standards, were purchased individually from AccuStandard (New Haven, CT, USA). All media used for extraction and clean-up were purchased from UTC (UTC Inc, Bristol, PA, USA).

All microplastics and algae with and without PCBs were extracted in triplicate using the QuEChERS method. Clams and sturgeon from each experimental tank were also extracted using the QuEChERS method. Extraction methods were developed based on Morrison et al. [43]. For microplastics, 2 grams were combined with 6 mL ultrapure water and spiked with 50 μL of 1 ng/μL internal standard. PCBs were extracted in 10 mL hexane using a vortex mixer for 2 minutes followed by a second extraction with 4 g of anhydrous MgSO_4_ with 1 g NaCl vortexed for 1 minute. Samples were centrifuged for 10 minutes at 3,000 RCF and entire extracts were transferred to a clean glass tube. For cleanup, 25 mg of a mixture of PSA/C18 media with 150 mg of anhydrous MgSO_4_ was added to the sample and vortexed for 1 minute. Samples were centrifuged for 10 minutes at 3,000 RCF and cleaned extracts were transferred to a clean glass tube. The cleanup process was repeated, and clean extracts were concentrated under nitrogen flow to 450 μL and spiked with 50 μL of 1 ng/μL recovery standard. For algae samples, 10 mL of sample (0.8 grams) was extracted using the same protocol without the addition of any water and using ethyl acetate as the extraction solvent. Clams from both experiments and sturgeon (with head and guts removed) were homogenized. Two samples of 10 clams, ranging from 3–12 grams each in total, from each tank were extracted using QuEChERS. Two sturgeon from each tank were extracted individually using QuEChERS. The extraction and cleanup methods described above for plastics and algae were used for animals with the exception of using ethyl acetate to extract via sonication for 30 minutes.

All glassware was cleaned and baked at 450°C for 5 hours. During extraction, samples were covered with aluminum foil to prevent contamination. Laboratory procedural blanks were extracted and run with every sequence of extracted samples. Blank levels measured in procedural blanks were subtracted from the reported concentrations of PCBs extracted from samples. Spiked matrix blanks were also extracted and run with every sequence of extracted samples. These were samples of baked NaSO_4_ spiked with known concentrations of all target analytes. In spiked matrix blank samples, recoveries of the four target analytes were greater than 81%. The recovery of the internal standards ranged from 70–82% for algae, 27–67% for microplastics, 32–64% for clams and 42–87% for sturgeon. The reported concentrations are recovery corrected based upon the recovery efficiencies of internal standards. Three quality control criteria were used to guarantee correct identification of target compounds: GC retention times matched those of standard compounds within ± 0.2 minutes, signal-to-noise ratio was greater than five, and the ratio between the quantitation and confirmation ions of each target compound was within ± 25% of the theoretical value.

All samples were analyzed for ^13^C PCBs using an Agilent 6890 series gas chromatograph and Agilent 5973 mass spectrometer (Agilent, Santa Clara, CA, U.S.) with ultrapure grade helium (99.995%: Airgas West El Cajon, CA, USA) as the carrier gas. Two μL of sample were injected into a Restek Rxi-5 Sil MS column (30 m × 0.25 mm i.d. × 0.25 μm thickness) integrated with a 5 m guard column. The oven program started at 75°C, held for 2 minutes, increased by 40°C per minute until 155°C, increased by 15°C and held for 3 minutes, increased by 3°C per minute until it reached 250°C and held for 5 minutes, increased by 50°C for 1 minute and increased to 320°C and held for 10 minutes. Selected ion monitoring (SIM) was used to detect 4 ^13^C PCBs (CB# 81, 77, 126 and 169), internal and recovery standards. The method quantification limit was 2 μg/L.

Because we could not detect PCBs in clams or sturgeon using the above instrumentation, clam samples and some sturgeon samples were also analyzed on an Agilent 7890N Gas Chromatograph (Palo Alto, USA) coupled to a Waters Xevo G2-XS atmospheric pressure chemical ionization (APCI) quadrupole time-of-flight mass spectrometer (qTOF, Waters, Manchester, UK). One μL of each sample was injected in pulsed splitless mode onto a 40m (0.18 mm i.d. x 0.18 μm) DB-5 column connected to a 0.8 m x 1.8 mm MXT (Restek, USA) deactivated stainless steel capillary column inserted into the transfer line. The program started at 80°C, held for 1 minute, then increased at 30°C per minute until it reached 210°C, then increased at 8°C per minute until it reached 320°C and then held for 5 minutes. The temperature of the injector was 250°C. Helium was used as the carrier gas at a constant flow of 1.5 mL/minute. The mass spectrometer was run in full scan mode (*m/z* 50–1000) with four separate target enhanced functions with a 1 second scan time centered on the M^+•^ ion for the two predominant ions of the targeted congeners (^13^C PCB# 81, 77, 126 and 169, plus internal and recovery standards). The corona voltage was set at 5 mAU, nitrogen was used for the auxiliary gas, cone gas and desolvation gas at 350 L/hour, 160 L/hour and 100 L/hour, respectively. The interface and ion source were held at 300°C and 150°C, respectively. A background ion present in the source region (*m/z* 212.0750) was used as an internal mass calibrant [44]. Mass accuracy was <2 ppm at *m/z* 359.8145 (PCB 155). Resolution (FWHM) was approximately 18,000 at the same *m/z*. Using dilutions of matrix standards to achieve lower concentrations of all target PCB analytes, we determined that method detection limits (MDL) for these samples ranged from 0.1 pg/g (parts per trillion) for PCB#126 and 169 to 0.2 pg/g (parts per trillion) for PCB# 81 and 77.

### Modelling the bioaccumulation of PCBs

The dynamic bioaccumulation model of Koelmans et al. [45] was used to estimate differences in the bioaccumulation of PCBs in clams exposed to different PCB-containing polymers. This approach is based on a mass balance of uptake and loss processes between ingested plastic, the gut contents, and organism tissues. The model allows for reversible transfer of PCBs between the gut and plastic over the gut retention time period. The direction of net transfer is determined by the relative concentrations of PCB in the gut and the plastic. Details of the parameters used (S1 Table) and modelling methodology are given in the Supplementary Information (S1 Methods).

### Immunohistochemistry

Fluorescence was measured on one random clam and the liver of one random fish from each tank (n = 3 animals per treatment). All enzyme substrates, protease inhibitors, salts and other chemicals were of analytical grade (Sigma Aldrich and ThermoFisher Scientific). Buffers were prepared using ultrapure water. The primary antibody used to measure vitellogenin in bivalves (anti-vitellogenin of *H*. *discus hannai*) was from Dr. Masahiko Awaji (National Research Institute of Aquaculture, Minami-Ise, Mie 516–0193, Japan). The primary antibody used to measure vitellogenin in fish was a monoclonal mouse anti-goat sturgeon vitellogenin antibody clone ND-1H2 (Biosense Laboratories). The primary antibody used to measure CYP450 (Polyclonal rabbit anti-fish CYP1A Peptide; Biosense Laboratories) and the secondary antibodies (Goat anti-rabbit IgG (H+L), Alexa Fluor® 488 and Goat anti-Rabbit IgG (H+L), Alexa Fluor® 555; Life Technologies) used to stain proteins were purchased from ThermoFisher Scientific.

Fixed samples of whole clams and the livers of fish were embedded in paraffin and sectioned at 5 μm. For clams, separate sections were used for CYP450 and vitellogenin. For fish, double staining on the same section was used for both antibodies. All sections were deparaffinized in citrisolv (Histo-Clear, National Diagnostics), rehydrated in graded ethanol solutions and rinsed in a Phosphate buffered saline (PBS) solution. Heat-induced antigen retrieval was performed in a 0.01 mol/L citrate buffer (pH = 6.0) antigen retrieval solution (DAKO antigen retrieval, Agilent Technologies, Santa Clara, CA, USA) for 30 minutes in a steamer. All sections were permeabilized for 45 minutes at room temperature in a 0.2% permeabilization detergent solution (Triton^TM^ X-100, Sigma-Aldrich) in PBS. Next, a signal enhancer (Image-iT^®^ FX, Life Technologies) was applied directly onto each section for 30 minutes at room temperature in a humid chamber. Sections were then pre-incubated in 10% bovine serum albumin (BSA, Roche Diagnostic) in PBS for one hour on a gentle rocker to block non-specific binding. Sections were then incubated overnight at 4°C with the following primary antibodies: Rabbit anti-fish CYP1A polyclonal, 1:50 dilution or anti-vitellogenin, 1:500 dilution. Next, the immunoreaction was developed using the secondary antibody Alexa Flour® 488 Rabbit (Life Technologies) with a 1:200 dilution applied in the dark and incubated for two hours at room temperature under humid conditions. Lastly, slides were mounted using mounting media with DAPI staining (ProLong® Diamond Antifade Mountant with DAPI, Life Technologies) and coverslipped. Slides were kept to dry for one hour at room temperature in the dark prior to taking photographs. As a negative control, the primary antibody was omitted from one test section on each day that we prepared samples for analysis.

Stained slides were photographed within 24 hours of staining using an Olympus DP71 camera coupled to an Olympus BX60 microscope with fluorescence filters. All photographs were taken under the same magnification (20×) and exposure setting. Using the negative control, the exposure setting was determined to eliminate background noise. For each section, five regions of each specific tissue of interest (digestive glands for CYP450 and gonads for vitellogenin) were photographed for each individual animal to get a representative assessment of the presence of CYP450 and vitellogenin antigens. For each region, two pictures were taken of the tissue of interest: one of the DAPI staining and another of the green fluorescence using the appropriate laser filters on the microscope. CYP450 and vitellogenin were quantified after digital capture using ImageJ software (National Institutes of Health, Bethesda, MD). Fluorescence was quantified from each picture using the same threshold function settings for all images. The threshold function was used to measure the brightness of fluorescent staining from proteins of interest, omitting blank space and non-targeted staining. The mean fluorescence intensity (counts/pixel) from each image was determined in ImageJ and the average intensity of the five images from each individual animal was calculated to quantify the immunofluorescence of CYP450 and vitellogenin for each individual animal.

### Histopathology

Individual clams and the livers and gastrointestinal tracts of fish were prepared for histopathological analysis to look for visible lesions or abnormalities. At the end of the experiment, three clams and four fish from each tank were randomly sampled, fixed in 10% neutral buffered formalin, dehydrated in a graded ethanol series and embedded in paraffin. Serial trans-sagittal sections (3 μm) were stained with hematoxylin and eosin (H&E). Lateral views of all samples were blindly screened using a BH-2 Olympus microscope. Histological lesions among individuals were rated on a scale from normal, mild, moderate to severe.

### Feeding behavior

For clams, all tanks were given their usual concentration of algae. Water samples were taken at 0 and 4 hours to measure the clearance rate via measurements of chlorophyll a. Water samples were filtered with 0.45μm glass fiber filters, extracted in a solution of 90:10 acetone:MgCO_3_ and measured via spectrophotometry. Clearance rate was calculated using the equation: clearance rate = (M/(n*t))*ln(C_0_/C_t_), where M is the volume of water in the tank, n is the number of clams, t is the time in minutes, C_0_ is the chlorophyll a concentration at time 0 and C_t_ is the chlorophyll a after 4 hours [46]. For fish, feeding behavior was calculated by quantifying the amount of food (by weight) ingested in each tank during a 1-minute time period. Equal amounts of diet were added to each tank. Remaining food was then siphoned out of the tank, filtered, dried and weighed. The final amount was subtracted from the initial amount to determine the amount of diet ingested per tank.

### Condition factor

After 28 days, six individual fish were measured and weighed to calculate condition factor. Condition factor (CF) was calculated using the equation CF = 100 × (weight g)/(length mm^3^) [47].

### Statistics

Differences in feeding behavior, CYP450 and vitellogenin protein expression, and histopathology among treatments were analyzed using 2-factor ANOVAs (n = 3, α = 0.05) with fixed factors “polymer” (5 levels: control, PET, polyethylene, PVC, polystyrene) and “PCBs” (2 levels: PCBs absent, PCBs present). For condition factor, six individual fish were measured from each tank. Differences among treatments were analyzed using a 3-factor ANOVA (n = 3, α = 0.05) with fixed-factors ‘‘polymer” and “PCBs” and random-factor ‘‘tank” nested in fixed factors ‘‘polymer” and “PCBs”. Because the p value for the nested term was > 0.250, this term was pooled. We assured that our data was normally distributed via histograms. We did not run statistical tests for normality because ANOVAs are not very sensitive to moderate deviations from normality [48]. To test for homogeneity of variance, we ran a Cochran’s C-test. For most of the data, a Cochran’s (1951) C-test (α = 0.05) showed homogeneity of variances. In two cases (vitellogenin for clams and condition factor for sturgeon), variances were heterogeneous, but still analyzed because analysis of variance is relatively robust to heterogeneous variances [48]. Student-Newman-Keuls (SNK) tests (α = 0.05) distinguished significantly different treatment means. Statistical analyses were performed using GMAV (EICC, University of Sydney).

## Results and discussion

### Bioaccumulation of PCBs

PCBs were not detected in any of the non-PCB treatment materials (i.e., algae, PET, polyethylene, PVC and polystyrene) except for a small concentration (3 ng/g) of PCB 126 in one replicate sample of algae. Because this was found in 1 of 3 replicates, this is likely due to column contamination in the GC/MS or cross-contamination during sample processing. In spiked samples, concentrations of PCBs in algae, polyethylene and polystyrene were one order of magnitude greater than in PET and PVC (Table 1; See S2 Table for all data). As such, we expected that concentrations of PCBs available for bioaccumulation would differ across polymers.

**Table 1:** PCBs in algae and plastics. Average measured concentrations (ng/g ± S.E.) of ^13^C PCBs in algae, PET, PVC, PE (polyethylene) and PS (polystyrene) used in experiments (n = 3). ND is non-detect.

Sample	PCB#81	PCB#77	PCB#126	PCB#169
**Algae**	ND	ND	1 ± 1	ND
**Algae + PCB**	18 ± 2	18 ± 2	19 ± 1	20 ± 1
**PET**	ND	ND	ND	ND
**PE**	ND	ND	ND	ND
**PVC**	ND	ND	ND	ND
**PS**	ND	ND	ND	ND
**PET + PCB**	8 ± 1	7 ± 1	7 ± 2	8 ± 1
**PE + PCB**	18 ± 0	18 ± 0	21 ± 1	18 ± 1
**PVC + PCB**	8 ± 1	8 ± 1	8 ± 1	8 ± 1
**PS + PCB**	14 ± 0	13 ± 0	14 ± 1	16 ± 1

After the 28-day exposure, across all samples of bivalves and fish, no ^13^C PCBs were detected above blank levels. This could be because no bioaccumulation occurred, concentrations were too low to detect by GC/MS, coplanar compounds were metabolized and excreted by clams, or that PCBs associated with microplastics were not available for uptake (e.g., due to volatilization of PCBs from microplastics from aeration in the tanks). Because our original objective was to measure differences in bioaccumulation among polymers, a biodynamic model developed by Koelmans et al. [45] was run to estimate what we could not measure.

The model predicted that, under our exposure conditions, clams would bioaccumulate PCBs and that concentrations should vary across polymers (S3 Table; S1 Fig). According to model predictions, concentrations of the sum of PCBs in clams after 28-days should have ranged from roughly 450–800 pg/g with concentrations being greatest for clams fed polyethylene and least in those fed PET (S3 Table; S1 Fig). Moreover, the model predicted that steady state would not have been reached during the 28-day experiment for any treatment. Predicted time to steady state ranged from roughly 130–300 days, varying by PCB congener and treatment (i.e., polymer or algae). Overall, predicted concentrations of total PCBs in clams after 28 days and at steady state were less for PET and PVC than for polyethylene and polystyrene. These predicted concentrations follow the same pattern as the sorption of PCBs to plastics, i.e. lower concentrations of PCBs on PET and PVC than polyethylene and polystyrene.

### Direct impacts to prey

**Fig 1:**
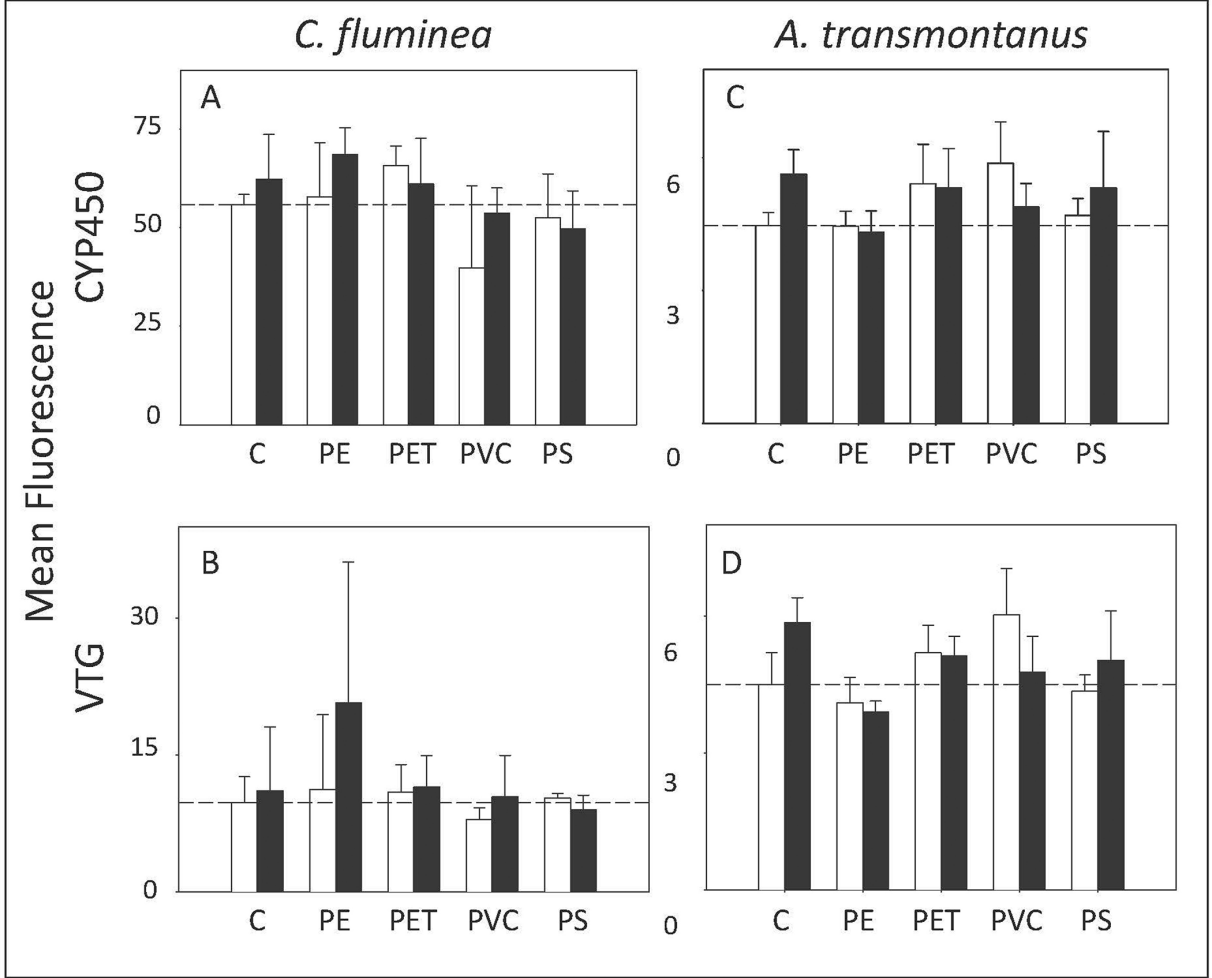
Immunohistochemistry. CYP450 (top) and vitellogenin (bottom) expression presented as mean fluorescence in clams (*C*. *fluminea*) (a, b) and fish (*A*. *transmontanus*) (c, d). Dotted lines represent the average value for the negative control treatment. Non-PCB treatments are depicted by white bars and PCB treatments are depicted by black bars (n = 3).

Immunohistochemistry can measure the presence of proteins produced due to the exposure to a chemical. Although the expression of CYP450 is well documented in the presence of dioxin-like coplanar PCBs to aid metabolism [28, 49], no significant differences across treatments for CYP450 were observed (n = 3; p > 0.05 for all factors; Fig 1A; See S4 Table for data).

Vitellogenin was measured in both species because a reduction in vitellogenin expression has been demonstrated in female fish exposed to PCBs and microplastics in our previous study [34]. For clams, there was no significant difference across treatments for vitellogenin (n = 3; p > 0.05 for all factors; Fig 1B; See S4 Table for data).

**Fig 2:**
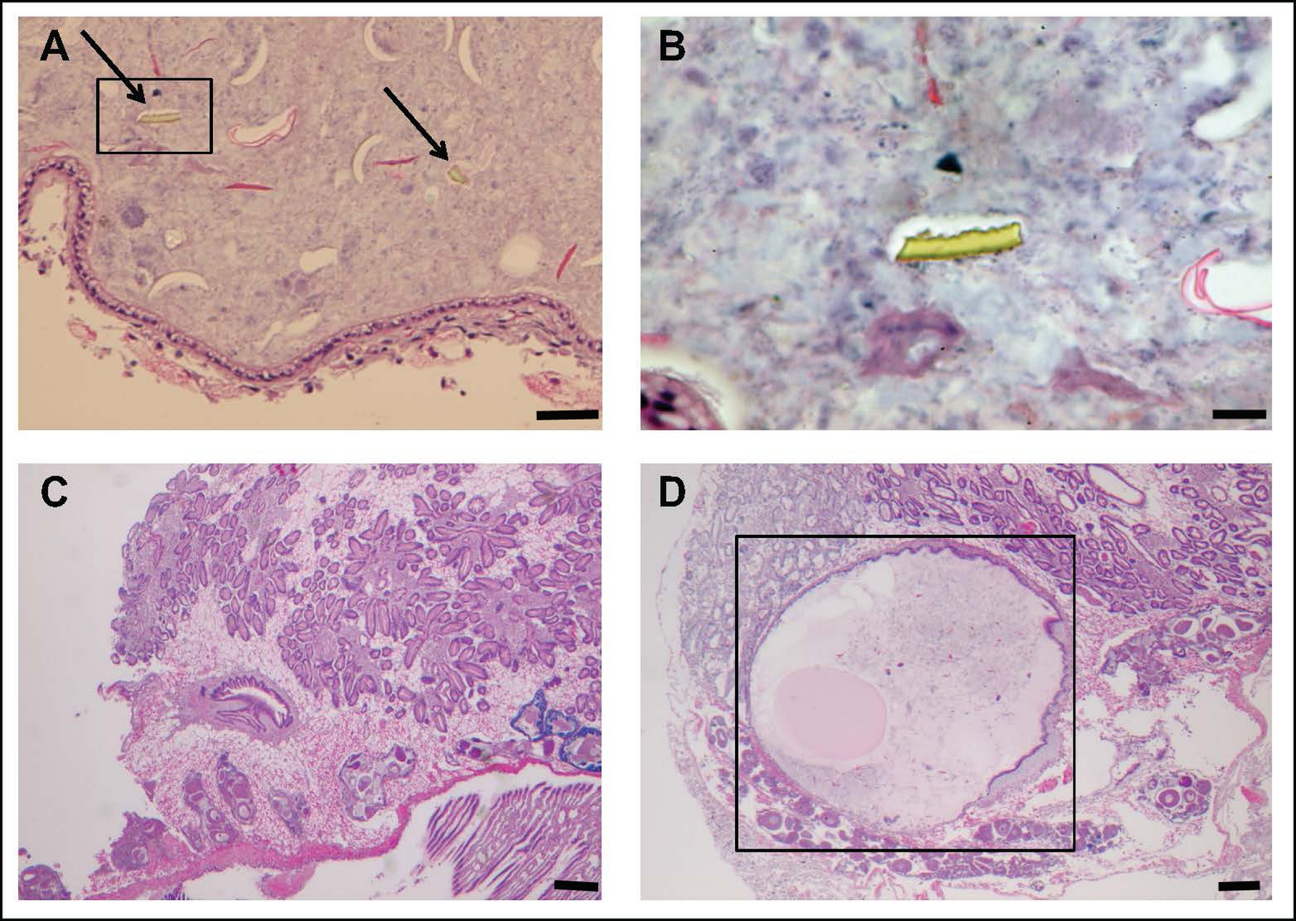
Histopathology sections. Histopathological sections of *C*. *fluminea* after the 28-day dietary exposure. Micrographs on the top show microplastic fragments in the gastrointestinal tract (a, depicted by arrows; scale bar = 50μm) and magnified (b; scale bar = 20μm). Micrographs on the bottom show a clam from the control treatment with a normal stomach (c; scale bar = 200μm) and a clam from the PVC+PCB treatment with a congested stomach and degeneration of the digestive gland (d; outlined in a box; scale bar = 200μm).

**Fig 3:**
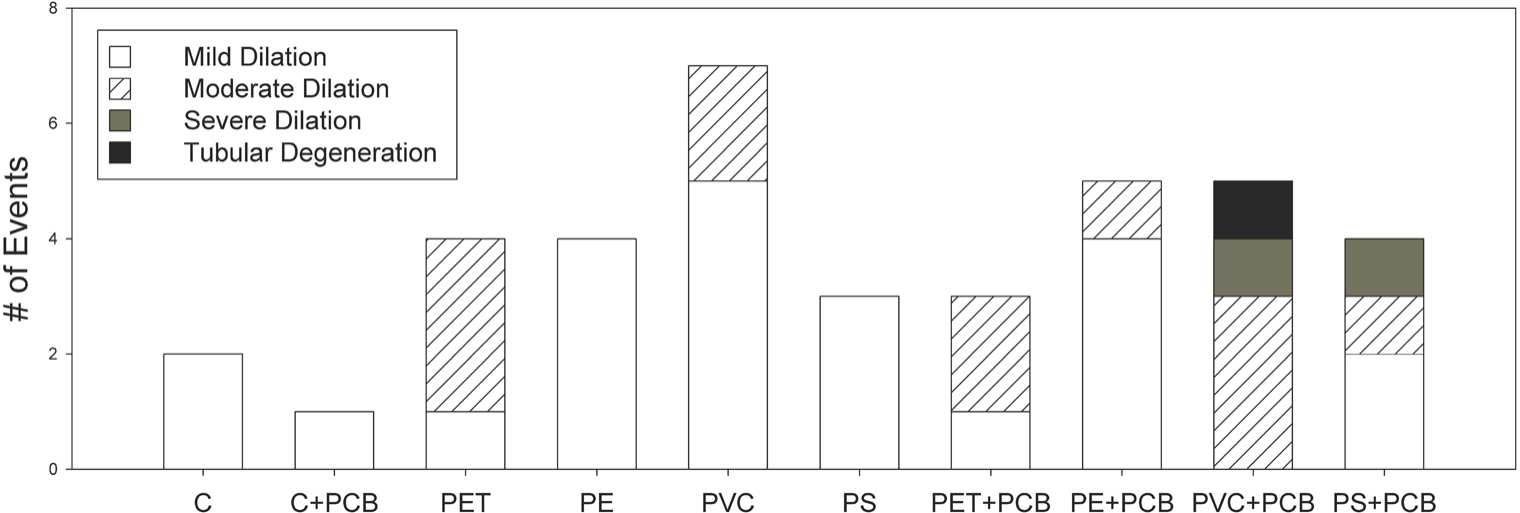
Histopathology abnormalities in clams. Number of histopathological abnormalities across all individual clams examined from each treatment (n = 9 clams per treatment) after the 28-day exposure. Individual bars represent the number of abnormalities (y axis) per treatment (x axis) and shading represents the type of abnormality: mild tubular dilation (white), moderate tubular dilation (striped), severe tubular dilation (dark grey) and tubular degeneration (black).

Histopathology was examined in sections of the whole body of clams to determine if exposure caused visible lesions or abnormalities. Microplastic pieces were observed in the stomachs of most clams exposed to microplastics (Fig 2A and 2B). This suggests the retention time of some microplastics may be longer than 48 hours. In clams, the only histological changes were seen in the digestive glands (Fig 2C and 2D). Overall, there were no differences in the numbers of histological abnormalities among treatments (n = 3; p > 0.05 for all factors). Out of the 90 clams examined, mild tubular dilation was observed in 23, moderate tubular dilation in 12 and severe tubular dilation in 2 clams with all moderate and severe effects seen in clams exposed to polymer and PCBs (See Fig 3 and S5 Table for detailed results regarding all individual clams from each treatment). In general, abnormalities did not seem to be caused by PCBs alone. On average, clams exposed to plastic had 3 times more abnormalities than clams that were not. Moreover, clams exposed to the combination of microplastic and PCBs had a greater occurrence of moderate and severe tubular dilation than clams fed microplastics only. Severe tubular dilation was seen in clams fed the combination of PCBs and PVC or polystyrene. Dilation appeared to be caused by the plastic, and enhanced by the combination of plastic and PCBs. Only one clam, from the PVC+PCB treatment, suffered tubular degeneration.

**Fig 4:**
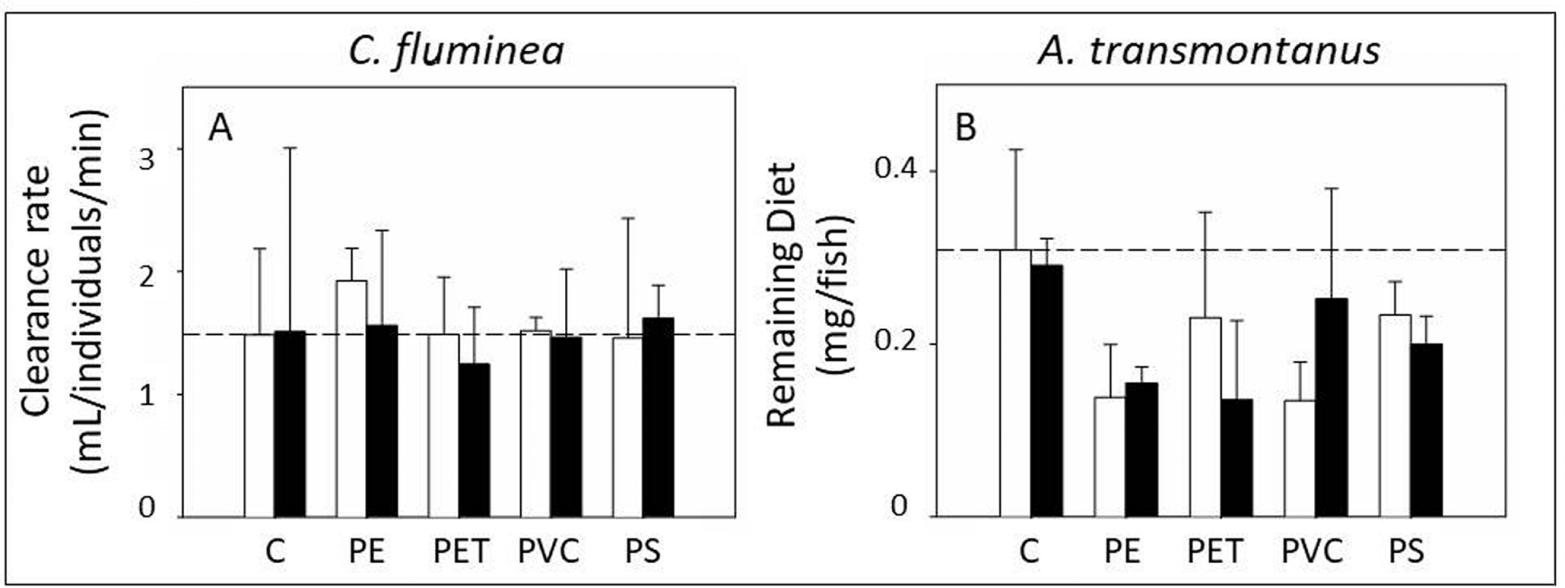
Feeding behavior. Feeding behavior expressed as clearance rate (mL/individuals/min) in clams (*C*. *fluminea*) (a) and remaining diet (mg/fish) in fish (*A*. *transmontanus*) (b). Dotted lines represent the average value for the negative control treatment. Non-PCB treatments are depicted by white bars and PCB treatments are depicted by black bars (n = 3).

The feeding behavior of clams was not significantly different amongst treatments (n = 3; p > 0.05 for all factors; Fig 4A; See S6 Table for data).

Mortality was minimal for clams. Mortality occurred in only four tanks, ranging from 3–10%. The remaining tanks had 100% survival during the entire exposure. There was no significant pattern among treatments (See S7 Table for data).

### Indirect impacts to predators

For fish, there was no significant difference in CYP450 amongst treatments (n = 3; p > 0.05 for all factors; Fig 1C; See S4 Table for data).

Vitellogenin levels did differ significantly amongst treatments due to polymer and the interaction between polymer and PCBs (n = 3; p = 0.015 and p = 0.0386 respectively; Fig 1D; See S4 Table for data). SNK comparisons did not reveal a significant pattern among treatments. This could be due to the high variability amongst replicates. Although post-hoc SNK tests could not differentiate amongst treatments, there was a trend where fish exposed to clams that were fed polyethylene (with and without PCBs) and polystyrene (without PCBs) had less vitellogenin proteins than fish fed clams from other treatments.

Histopathology was examined in the livers and gastrointestinal tracts of fish. Glycogen depletion was observed in only 3 fish. Severe glycogen depletion was observed in 1 fish from the PVC+PCB treatment and moderate glycogen depletion in 1 fish from the PET+PCB and 1 fish from the polystyrene treatment.

Sturgeon exhibited differences in feeding behavior when exposed to clams fed microplastic. The difference was only significant for the factor polymer and not PCBs (n = 3; p = 0.0175; Fig 4B; See S6 Table for data). SNK comparisons could not reveal a significant pattern among treatments. This could be due to the high variability amongst replicates. Still, there was a trend where fish exposed to microplastics ingested more of their diet than fish that were not exposed to microplastics. These effects seemed to be caused by the microplastic itself rather than the PCBs or a mixture effect of plastic and PCBs together. Other studies have observed changes in feeding behavior as well, but results are mixed. While many studies have observed decreases in feeding after exposure to microplastics [50, 51], another study observed an increase in feeding [11]. They found that oysters, exposed to microplastics, ate more of their algal diet. Similarly, we found that fish exposed to clams fed microplastics, consumed more of their diet.

Condition factor was also measured at the end of the 28-day exposure. Condition factors differed amongst the 10 treatments and the difference was only significant for the factor polymer (n = 3; p = 0.035; See S8 Table for data). SNK comparisons did not reveal a significant pattern among polymer treatments and there was no apparent trend.

Fish mortality was equal to zero.

## Conclusions

Experiments using environmentally relevant concentrations are necessary to understand the implications of the widespread contamination of microplastics in aquatic habitats. The environmental relevance of this study included exposures with freshwater prey and predator and to a relatively realistic dose of common types and sizes of microplastics with commonly occurring PCBs. Here, exposures to microplastics caused subtle, direct effects in prey and subtle, indirect effects in predators. If plastic production and waste management trends continue as predicted, concentrations in aquatic habitats will likely increase [52]. Thus, further studies measuring impacts with current and projected future concentrations of microplastics in especially urban-impacted freshwaters, are necessary.

Furthermore, experiments that test different shapes (e.g., microfibers) and types of microplastics are useful. Microplastics come in a variety of polymers and shapes, some of which are relatively benign and others that have hazardous constituent monomers or additives [20]. In our study, we used several different plastics that were similar in shape and size and we found some effects varied by polymer type. For example, histological abnormalities were greatest in clams fed PCBs and PVC or polystyrene. Just like chemical pollutants, not all microplastics should be treated equally and/or categorized into a homogenous contaminant group. Instead of only thinking about concentrations of microplastics that may be hazardous, we might also consider different sources and types of microplastics that may be hazardous. This may help ease some of the pressure on both managers and industry groups.

## Supporting information

S1 Methods Methods related to the bioaccumulation model. (DOCX)

S1 Table Modelling parameters used in the bioaccumulation model. (DOCX)

S2 Table Concentrations of PCBs in algae and plastic samples after being spiked. Concentrations are in ng/g and are provided for negative treatments (no PCBs) and positive treatments (with PCBs). Concentrations are not provided for clams and sturgeon because they are all ND.
(DOCX)

S3 Table Concentrations (pg/g) of ^13^C PCBs in Asian clam calculated by the model. Calculated concentrations (pg/g) of ^13^C PCBs in Asian clam (Cb) fed algae, PET, PVC, PE (polyethylene) and PS (polystyrene) with PCBs after 28 days and at steady state based on model predictions. Days to steady state is shown in parentheses after each concentration in the tissues.
(DOCX)

S4 Table Immunohistochemistry data for clams and sturgeon. Immunohistochemistry data: protein expression expressed as fluorescence for clams and sturgeon for both vitellogenin (VTG) and cytochrome P450 (CYP1a).
(DOCX)

S5 Table Histological data of tubular dilation observed in the histology slides of Asian clams. Histopathology data for Asian Clams showing tubular dilation observed in the histology slides (n = 3, 9 individual clams per treatment). The second column provides the number of clams with dilation vs. non-dilation. The third column provides the severity of the clams with dilation on a scale from mild, moderate to severe.
(DOCX)

S6 Table Feeding behavior assay data for clams and sturgeon. Feeding behavior assay data for clams and sturgeon. For clams, data is expressed as clearance rate (mL/individual/minute). For sturgeon, data is expressed as diet remaining in the tank (mg).
(DOCX)

S7 Table Mortality data for clams. Mortality measured in clams over the entire 28-day exposure.
(DOCX)

S8 Table Condition factor data for sturgeon. Condition factors measured in six individual fish at the end of the 28-day exposure.
(DOCX)

S1 Fig Model predictions. A graph showing model predictions across polymers.
(DOCX)
