# Supplementary material for: Direct and indirect effects of different types of microplastics on freshwater prey (Corbicula fluminea) and their predator (Acipenser transmontanus)
Source: PLoS One. 2017 Nov 6;12(11):e0187664. doi: 10.1371/journal.pone.0187664 (PMC5673206; doi:10.1371/journal.pone.0187664)
Supplement: S1 Methods — (DOCX) [file pone.0187664.s001.docx]

**S1 Methods.** Description of the bioaccumulation model.

Modelling Details:

The Koelmans et al. [43] model is designed to allow for chemical transfer to the organism directly from the surrounding water, and from ingested sediment.  In our study, the model was simplified by setting all sediment-related parameters and the background water concentration to 0, as appropriate for the present work. Rate coefficients for metabolism and excretion (k_loss_) processes were based on those of PCBs with similar partition coefficients (PCB52 for PCB77 and 81, PCB118 for PCB126, and PCB153 for PCB169; [44]). The remaining modelling parameters were set to match the conditions of our experiment, as summarized in Table 1. Using a time step of one day, the model was run with two different sets of input plastic-water partition coefficients and assuming that spiked PCBs had reached equilibrium with the plastic within the 2-week period. One version used plastic-water partition coefficients (see Table 1) that were estimated using COSMO-RS theory, as implemented in the COSMOtherm v 16.01 program [45, 46], to derive the lipid-to-plastic transfer rate coefficient; the other version used plastic-water partition coefficients estimated based on empirical measurements from Rochman et al. [21] and calculated by Endo and Koelmans [47] under the assumption that the partition coefficients of polystyrene and polyethylene were similar based on empirical data from Rochman and Monzano et al. [48].
