# Supplementary material for: Direct and indirect effects of different types of microplastics on freshwater prey (Corbicula fluminea) and their predator (Acipenser transmontanus)
Source: PLoS One. 2017 Nov 6;12(11):e0187664. doi: 10.1371/journal.pone.0187664 (PMC5673206; doi:10.1371/journal.pone.0187664)
Supplement: S1 Table — (DOCX) [file pone.0187664.s002.docx]

**S1 Table. Definition and units of model parameters for bioaccumulation model.**

| **Parameter** | **Definitions and units** | **Source of model input** |
| --- | --- | --- |
| **k_1_** | Rate constant for chemical release from plastic in GIT (-d) | 2.1 (Koelmans et al., 2016 ES&T) |
| **C_PL_** | Concentration in plastic (ng/g) | Measured value from experiment |
| **M_PL_** | Mass of plastic (g) | Measured value from experiment |
| **GRT** | Gut residence time (days) | 1.25 days (Decho and Luoma, 1991) |
| **IR** | Ingestion rate (g/g WW x –d) | Measured value from experiment |
| **S(PL)** | Mass fraction of plastic | Measured value from experiment |
| **k_loss_** | Loss rate into water (-d) | 0.04 PCB 77, 81 (based off of PCB52) and 0.03 PCB 126, 169 (based off of PCB118 and PCB153; Boese et al., 1997 ET&C) |
| **f(lipid)** | Lipid fraction | 0.182 (Chijimatsu et al., 2011 British Journal of Nutrition) |
| **C_b(0)_** | Concentration of PCBs in animal at day 0 (ng/g) | Measured value from experiment |
| **w** | species wet weight (g) | Measured value from experiment |
| **Log K_ow_** | log octanol-water partition ratio | Lohmann, 2012 ES&T |
| **Log K_pw_** | log plastic-water partition coefficient | Calculated using COSMOtherm & Koelmans et al., 2016 |
