# Supplementary material for: Direct and indirect effects of different types of microplastics on freshwater prey (Corbicula fluminea) and their predator (Acipenser transmontanus)
Source: PLoS One. 2017 Nov 6;12(11):e0187664. doi: 10.1371/journal.pone.0187664 (PMC5673206; doi:10.1371/journal.pone.0187664)
Supplement: S2 Table — Concentrations are in ng/g and are provided for negative treatments (no PCBs) and positive treatments (with PCBs). Concentrations are not provided for clams and sturgeon because they are all ND. (DOCX) [file pone.0187664.s003.docx]

**S2 Table. Concentrations of PCBs in algae and plastic samples after being spiked.** Concentrations on are ng/g and are provided for negative treatments (no PCBs) and positive treatments (with PCBs). Concentrations are not provided for clams and sturgeon because they are all ND.

| **Sample ID** | **PCB 81 (ng/g)** | **PCB 77 (ng/g)** | **PCB 126 (ng/g)** | **PCB 169 (ng/g)** |
| --- | --- | --- | --- | --- |
| Algae -C Rep 1 | ND | ND | ND | ND |
| Algae -C Rep 2 | ND | ND | 3 | 0 |
| Algae -C Rep 3 | ND | ND | ND | ND |
| Algae +C Rep 1 | 17 | 20 | 17 | 19 |
| Algae +C Rep 2 | 16 | 15 | 20 | 19 |
| Algae +C Rep 3 | 22 | 18 | 20 | 21 |
| -PET 1 | ND | ND | ND | ND |
| -PET 2 | ND | ND | ND | ND |
| -PET 3 | ND | ND | ND | ND |
| -PE 1 | ND | ND | ND | ND |
| -PE 2 | ND | ND | ND | ND |
| -PE 3 | ND | ND | ND | ND |
| -PVC 1 | ND | ND | ND | ND |
| -PVC 2 | ND | ND | ND | ND |
| -PVC 3 | ND | ND | ND | ND |
| -PS 1 | ND | ND | ND | ND |
| -PS 2 | ND | ND | ND | ND |
| -PS 3 | ND | ND | ND | ND |
| +PET 1 | 10 | 10 | 10 | 11 |
| +PET 2 | 8 | 7 | 8 | 8 |
| +PET 3 | 6 | 5 | 5 | 6 |
| +PE 1 | 18 | 18 | 19 | 16 |
| +PE 2 | 19 | 18 | 21 | 20 |
| +PE 3 | 19 | 18 | 21 | 18 |
| +PVC 1 | 7 | 7 | 6 | 7 |
| +PVC 2 | 10 | 10 | 10 | 9 |
| +PVC 3 | 8 | 7 | 8 | 9 |
| +PS 1 | 14 | 13 | 15 | 15 |
| +PS 2 | 15 | 13 | 13 | 15 |
| +PS 3 | 14 | 13 | 14 | 17 |
