# Supplementary material for: Direct and indirect effects of different types of microplastics on freshwater prey (Corbicula fluminea) and their predator (Acipenser transmontanus)
Source: PLoS One. 2017 Nov 6;12(11):e0187664. doi: 10.1371/journal.pone.0187664 (PMC5673206; doi:10.1371/journal.pone.0187664)
Supplement: S3 Table — Calculated concentrations (pg/g) of 13C PCBs in Asian clam (Cb) fed algae, PET, PVC, PE (polyethylene) and PS (polystyrene) with PCBs after 28 days and at steady state based on model predictions. Days to steady state is shown in parentheses after each concentration in the tissues. (DOCX) [file pone.0187664.s004.docx]

**S3 Table.** Calculated concentrations (pg/g) of ^13^C PCBs in Asian clam (Cb) fed algae, PET, PVC, PE (polyethylene) and PS (polystyrene) with PCBs after 28 days and at steady state based on model predictions. Days to steady state is shown in parentheses after each concentration in the tissues.

| **PCBs** | **Model run using Kpws calculated using COSMOtherm** | | | | | | | | | |
| --- | --- | --- | --- | --- | --- | --- | --- | --- | --- | --- |
|  | Cb(28 d) pg/g | | | | | Cb(steady state) pg/g (days to steady state) | | | | |
|  | PET | PE | PVC | PS | Algae | PET | PE | PVC | PS | Algae |
| 77 | 115 | 179 | 116 | 152 | 97 | 167 (134) | 263 (134) | 169 (130) | 223 (159) | 142 (133) |
| 81 | 99 | 179 | 116 | 141 | 97 | 139 (133) | 262 (137) | 169 (140) | 207 (177) | 142 (133) |
| 126 | 113 | 235 | 130 | 171 | 167 | 193 (211) | 408 (206) | 225 (196) | 297 (232) | 291 (203) |
| 169 | 129 | 201 | 130 | 195 | 176 | 219 (187) | 350 (224) | 225 (208) | 339 (199) | 307 (265) |
| *ΣPCBs* | *456* | *794* | *492* | *659* | *537* | *742* | *1283* | *788* | *1066* | *882* |
| **PCBs** | **Model run using Kpws calculated based by Endo and Koelmans et al. (2016)** | | | | | | | | | |
|  | Cb(28 d) pg/g | | | | | Cb(steady state) pg/g (days to steady state) | | | | |
|  | PET | PE | PVC | PS | Algae | PET | PE | PVC | PS | Algae |
| 77 | 116 | 179 | 116 | 152 | 97 | 171 (173) | 262 (138) | 171 (173) | 224 (250) | 142 (133) |
| 81 | 102 | 179 | 116 | 142 | 97 | 149 (128) | 262 (138) | 171 (173) | 208 (208) | 142 (133) |
| 126 | 114 | 235 | 131 | 171 | 167 | 199 (192) | 409 (297) | 227 (178) | 298 (196) | 291 (203) |
| 169 | 131 | 201 | 131 | 196 | 176 | 227 (179) | 350 (231) | 227 (179) | 340 (192) | 307 (265) |
| *ΣPCBs* | *463* | *794* | *494* | *661* | *537* | *746* | *1283* | *796* | *1070* | *882* |
