# Supplementary material for: Direct and indirect effects of different types of microplastics on freshwater prey (Corbicula fluminea) and their predator (Acipenser transmontanus)
Source: PLoS One. 2017 Nov 6;12(11):e0187664. doi: 10.1371/journal.pone.0187664 (PMC5673206; doi:10.1371/journal.pone.0187664)
Supplement: S4 Table — Immunohistochemistry data: protein expression expressed as fluorescence for clams and sturgeon for both vitellogenin (VTG) and cytochrome P450 (CYP1a). (DOCX) [file pone.0187664.s005.docx]

**S4 Table.** Immunohistochemistry data: protein expression expressed as fluorescence for clams and sturgeon for both vitellogenin (VTG) and cytochrome P450 (CYP1a).

| **Treatment** | **Clams** | **fluorescence VTG** | **fluorescence CYP1a** | **Sturgeon** | **fluorescence VTG** | **fluorescence CYP1a** |
| --- | --- | --- | --- | --- | --- | --- |
| control |  | 7.0608 | 58.4702 |  | 4.50 | 4.44 |
| control |  | 12.7106 | 53.2436 |  | 3.79 | 4.20 |
| control |  | 9.557 | 55.752 |  | 5.20 | 4.78 |
| control+PCB |  | 17.4012 | 49.6422 |  | 6.45 | 6.23 |
| control+PCB |  | 3.489 | 66.7188 |  | 5.34 | 5.50 |
| control+PCB |  | 12.3104 | 70.8372 |  | 5.75 | 5.14 |
| PS |  | 9.7518 | 62.5832 |  | 3.95 | 4.27 |
| PS |  | 10.289 | 40.486 |  | 4.64 | 5.02 |
| PS |  | 10.7724 | 54.4968 |  | 4.46 | 4.79 |
| PS+PCB |  | 7.1798 | 38.7854 |  | 4.51 | 4.56 |
| PS+PCB |  | 10.111 | 56.9076 |  | 4.31 | 4.60 |
| PS+PCB |  | 9.7212 | 53.4458 |  | 6.27 | 6.79 |
| PET |  | 9.3946 | 71.1388 |  | 4.54 | 4.80 |
| PET |  | 14.4298 | 61.3668 |  | 5.33 | 4.99 |
| PET |  | 8.9308 | 64.9796 |  | 5.71 | 6.44 |
| PET+PCB |  | 11.4296 | 72.1802 |  | 5.62 | 5.76 |
| PET+PCB |  | 14.958 | 62.06 |  | 4.83 | 4.29 |
| PET+PCB |  | 8.0816 | 49.082 |  | 4.94 | 5.89 |
| PE |  | 8.9842 | 66.1044 |  | 3.59 | 4.10 |
| PE |  | 20.2834 | 42.1088 |  | 4.68 | 4.76 |
| PE |  | 4.2756 | 65.462 |  | 4.02 | 4.50 |
| PE+PCB |  | 38.4094 | 71.315 |  | 3.69 | 3.77 |
| PE+PCB |  | 13.3764 | 61.0158 |  | 4.15 | 4.71 |
| PE+PCB |  | 10.374 | 73.603 |  | 3.87 | 4.45 |
| PVC |  | 7.446 | 53.4218 |  | 5.68 | 5.70 |
| PVC |  | 9.4348 | 15.9582 |  | 7.16 | 6.88 |
| PVC |  | 7.0404 | 50.248 |  | 5.22 | 5.05 |
| PVC+PCB |  | 14.0152 | 58.5176 |  | 5.62 | 5.48 |
| PVC+PCB |  | 11.9198 | 56.148 |  | 4.09 | 4.48 |
| PVC+PCB |  | 5.289 | 46.3612 |  | 4.60 | 4.71 |
