# Supplementary material for: Direct and indirect effects of different types of microplastics on freshwater prey (Corbicula fluminea) and their predator (Acipenser transmontanus)
Source: PLoS One. 2017 Nov 6;12(11):e0187664. doi: 10.1371/journal.pone.0187664 (PMC5673206; doi:10.1371/journal.pone.0187664)
Supplement: S5 Table — Histopathology data for Asian Clams showing tubular dilation observed in the histology slides (n = 3, 9 individual clams per treatment). The second column provides the number of clams with dilation vs. non-dilation. The third column provides the severity of the clams with dilation on a scale from mild, moderate to severe. (DOCX) [file pone.0187664.s006.docx]

**S5 Table.** Histopathology data for Asian Clams showing tubular dilation observed in the histology slides (n=3, i.e., 9 individual clams per treatment). The second column provides the number of clams with dilation vs. non-dilation. The third column provides the severity of the clams with dilation on a scale from mild, moderate to severe.

| **Treatment** | **Normal** | **Dilation** | **Mild** | **Moderate** | **Severe** |
| --- | --- | --- | --- | --- | --- |
| **Neg Control** | 7 | 2 | 2 | 0 | 0 |
| **Positive Control** | 8 | 1 | 1 | 0 | 0 |
| **PET** | 5 | 4 | 1 | 3 | 0 |
| **PE** | 5 | 4 | 4 | 0 | 0 |
| **PVC** | 2 | 7 | 5 | 2 | 0 |
| **PS** | 6 | 3 | 3 | 0 | 0 |
| **PET + PCB** | 6 | 8 | 6 | 2 | 0 |
| **PE + PCB** | 4 | 5 | 4 | 1 | 0 |
| **PVC + PCB** | 5 | 4 | 0 | 3 | 1 |
| **PS + PCB** | 5 | 3 | 2 | 1 | 0 |
