# Supplementary material for: Direct and indirect effects of different types of microplastics on freshwater prey (Corbicula fluminea) and their predator (Acipenser transmontanus)
Source: PLoS One. 2017 Nov 6;12(11):e0187664. doi: 10.1371/journal.pone.0187664 (PMC5673206; doi:10.1371/journal.pone.0187664)
Supplement: S6 Table — Feeding behavior assay data for clams and sturgeon. For clams, data is expressed as clearance rate (mL/individual/minute). For sturgeon, data is expressed as diet remaining in the tank (mg). (DOCX) [file pone.0187664.s007.docx]

**S6 Table.** Feeding behavior assay data for clams and sturgeon. For clams, data is expressed as clearance rate (mL/individual/minute). For sturgeon, data is expressed as diet remaining in the tank (mg).

| **Treatment** | **Clams** | **Clearance Rate (ml/indv/min)** | **Sturgeon** | **Remaining Diet (mg)** |
| --- | --- | --- | --- | --- |
| Control |  | 2.26 |  | 0.336 |
| Control |  | 0.90 |  | 0.181 |
| Control |  | 1.29 |  | 0.409 |
| Control+PCB |  | 3.23 |  | 0.263 |
| Control+PCB |  | 0.81 |  | 0.325 |
| Control+PCB |  | 0.50 |  | 0.2847 |
| PET |  | 1.65 |  | 0.142 |
| PET |  | 1.85 |  | 0.37 |
| PET |  | 0.96 |  | 0.18 |
| PET+PCB |  | 1.76 |  | 0.064 |
| PET+PCB |  | 0.87 |  | 0.239 |
| PET+PCB |  | 1.11 |  | 0.103 |
| PE |  | 1.93 |  | 0.154 |
| PE |  | 2.18 |  | 0.071 |
| PE |  | 1.66 |  | 0.19 |
| PE+PCB |  | 2.45 |  | 0.175 |
| PE+PCB |  | 1.19 |  | 0.138 |
| PE+PCB |  | 1.05 |  | 0.152 |
| PVC |  | 1.42 |  | 0.148 |
| PVC |  | 1.51 |  | 0.085 |
| PVC |  | 1.63 |  | 0.171 |
| PVC+PCB |  | 0.83 |  | 0.263 |
| PVC+PCB |  | 1.87 |  | 0.374 |
| PVC+PCB |  | 1.69 |  | 0.12 |
| PS |  | 0.35 |  | 0.221 |
| PS |  | 1.87 |  | 0.204 |
| PS |  | 2.16 |  | 0.277 |
| PS+PCB |  | 1.41 |  | 0.198 |
| PS+PCB |  | 1.92 |  | 0.168 |
| PS+PCB |  | 1.52 |  | 0.233 |
