# Supplementary material for: Direct and indirect effects of different types of microplastics on freshwater prey (Corbicula fluminea) and their predator (Acipenser transmontanus)
Source: PLoS One. 2017 Nov 6;12(11):e0187664. doi: 10.1371/journal.pone.0187664 (PMC5673206; doi:10.1371/journal.pone.0187664)
Supplement: S7 Table — Mortality measured in clams over the entire 28-day exposure. (DOCX) [file pone.0187664.s008.docx]

**S7 Table.** Mortality measured in clams over the entire 28-day exposure period.

| **Treatment** | **Mortality** |
| --- | --- |
| Control | 0 |
| Control | 0 |
| Control | 0 |
| Control+PCB | 0 |
| Control+PCB | 3 |
| Control+PCB | 0 |
| PS | 0 |
| PS | 0 |
| PS | 0 |
| PS+PCB | 0 |
| PS+PCB | 0 |
| PS+PCB | 0 |
| PET | 1 |
| PET | 0 |
| PET | 0 |
| PET+PCB | 0 |
| PET+PCB | 0 |
| PET+PCB | 0 |
| PE | 0 |
| PE | 0 |
| PE | 0 |
| PE+PCB | 0 |
| PE+PCB | 1 |
| PE+PCB | 0 |
| PVC | 1 |
| PVC | 0 |
| PVC | 0 |
| PVC+PCB | 0 |
| PVC+PCB | 0 |
| PVC+PCB | 0 |
