# Supplementary material for: Direct and indirect effects of different types of microplastics on freshwater prey (Corbicula fluminea) and their predator (Acipenser transmontanus)
Source: PLoS One. 2017 Nov 6;12(11):e0187664. doi: 10.1371/journal.pone.0187664 (PMC5673206; doi:10.1371/journal.pone.0187664)
Supplement: S8 Table — Condition factors measured in six individual fish at the end of the 28-day exposure. (DOCX) [file pone.0187664.s009.docx]

**S8 Table.** Condition factors measured in six individual fish at the end of the 28-day exposure.

| **Treatment** | **fish** | **CF** | **Treatment** | **fish** | **CF** | **Treatment** | **fish** | **CF** |
| --- | --- | --- | --- | --- | --- | --- | --- | --- |
| C | 1 | 0.319 | PET+PCB | 1 | 0.361 | PVC | 1 | 0.375 |
| C | 2 | 0.312 | PET+PCB | 2 | 0.328 | PVC | 2 | 0.384 |
| C | 3 | 0.352 | PET+PCB | 3 | 0.349 | PVC | 3 | 0.388 |
| C | 4 | 0.330 | PET+PCB | 4 | 0.372 | PVC | 4 | 0.387 |
| C | 5 | 0.352 | PET+PCB | 5 | 0.344 | PVC | 5 | 0.380 |
| C | 6 | 0.329 | PET+PCB | 6 | 0.335 | PVC | 6 | 0.381 |
| C | 1 | 0.363 | PET+PCB | 1 | 0.325 | PVC | 1 | 0.337 |
| C | 2 | 0.315 | PET+PCB | 2 | 0.330 | PVC | 2 | 0.344 |
| C | 3 | 0.346 | PET+PCB | 3 | 0.321 | PVC | 3 | 0.350 |
| C | 4 | 0.350 | PET+PCB | 4 | 0.336 | PVC | 4 | 0.310 |
| C | 5 | 0.347 | PET+PCB | 5 | 0.310 | PVC | 5 | 0.385 |
| C | 6 | 0.321 | PET+PCB | 6 | 0.394 | PVC | 6 | 0.385 |
| C | 1 | 0.348 | PET+PCB | 1 | 0.375 | PVC | 1 | 0.385 |
| C | 2 | 0.353 | PET+PCB | 2 | 0.328 | PVC | 2 | 0.344 |
| C | 3 | 0.361 | PET+PCB | 3 | 0.373 | PVC | 3 | 0.379 |
| C | 4 | 0.360 | PET+PCB | 4 | 0.368 | PVC | 4 | 0.396 |
| C | 5 | 0.357 | PET+PCB | 5 | 0.364 | PVC | 5 | 0.352 |
| C | 6 | 0.317 | PET+PCB | 6 | 0.357 | PVC | 6 | 0.368 |
| C+PCB | 1 | 0.357 | PE | 1 | 0.409 | PVC+PCB | 1 | 0.392 |
| C+PCB | 2 | 0.347 | PE | 2 | 0.312 | PVC+PCB | 2 | 0.378 |
| C+PCB | 3 | 0.349 | PE | 3 | 0.341 | PVC+PCB | 3 | 0.384 |
| C+PCB | 4 | 0.373 | PE | 4 | 0.348 | PVC+PCB | 4 | 0.369 |
| C+PCB | 5 | 0.376 | PE | 5 | 0.387 | PVC+PCB | 5 | 0.377 |
| C+PCB | 6 | 0.342 | PE | 6 | 0.297 | PVC+PCB | 6 | 0.358 |
| C+PCB | 1 | 0.354 | PE | 1 | 0.320 | PVC+PCB | 1 | 0.354 |
| C+PCB | 2 | 0.334 | PE | 2 | 0.353 | PVC+PCB | 2 | 0.354 |
| C+PCB | 3 | 0.456 | PE | 3 | 0.372 | PVC+PCB | 3 | 0.396 |
| C+PCB | 4 | 0.346 | PE | 4 | 0.338 | PVC+PCB | 4 | 0.383 |
| C+PCB | 5 | 0.356 | PE | 5 | 0.345 | PVC+PCB | 5 | 0.331 |
| C+PCB | 6 |  | PE | 6 | 0.371 | PVC+PCB | 6 | 0.365 |
| C+PCB | 1 | 0.359 | PE | 1 | 0.459 | PVC+PCB | 1 | 0.344 |
| C+PCB | 2 | 0.338 | PE | 2 | 0.359 | PVC+PCB | 2 | 0.331 |
| C+PCB | 3 | 0.384 | PE | 3 | 0.391 | PVC+PCB | 3 | 0.382 |
| C+PCB | 4 | 0.397 | PE | 4 | 0.373 | PVC+PCB | 4 | 0.371 |
| C+PCB | 5 | 0.328 | PE | 5 | 0.325 | PVC+PCB | 5 | 0.406 |
| C+PCB | 6 | 0.383 | PE | 6 | 0.374 | PVC+PCB | 6 | 0.362 |
|  |  |  |  |  |  |  |  |  |
| **Treatment** | **fish** | **CF** | **Treatment** | **fish** | **CF** | **Treatment** | **fish** | **CF** |
| PET | 1 | 0.334 | PE+PCB | 1 | 0.384 | PS | 1 | 0.330 |
| PET | 2 | 0.343 | PE+PCB | 2 | 0.360 | PS | 2 | 0.365 |
| PET | 3 | 0.375 | PE+PCB | 3 | 0.369 | PS | 3 | 0.324 |
| PET | 4 | 0.359 | PE+PCB | 4 | 0.358 | PS | 4 | 0.365 |
| PET | 5 | 0.314 | PE+PCB | 5 | 0.346 | PS | 5 | 0.359 |
| PET | 6 | 0.333 | PE+PCB | 6 | 0.310 | PS | 6 | 0.287 |
| PET | 1 | 0.353 | PE+PCB | 1 | 0.322 | PS | 1 | 0.370 |
| PET | 2 | 0.318 | PE+PCB | 2 | 0.331 | PS | 2 | 0.325 |
| PET | 3 | 0.350 | PE+PCB | 3 | 0.333 | PS | 3 | 0.326 |
| PET | 4 | 0.310 | PE+PCB | 4 | 0.351 | PS | 4 | 0.338 |
| PET | 5 | 0.332 | PE+PCB | 5 | 0.414 | PS | 5 | 0.364 |
| PET | 6 | 0.384 | PE+PCB | 6 | 0.355 | PS | 6 | 0.347 |
| PET | 1 | 0.357 | PE+PCB | 1 | 0.357 | PS | 1 | 0.359 |
| PET | 2 | 0.365 | PE+PCB | 2 | 0.394 | PS | 2 | 0.358 |
| PET | 3 | 0.342 | PE+PCB | 3 | 0.381 | PS | 3 | 0.331 |
| PET | 4 | 0.369 | PE+PCB | 4 | 0.344 | PS | 4 | 0.360 |
| PET | 5 | 0.354 | PE+PCB | 5 | 0.387 | PS | 5 | 0.367 |
| PET | 6 | 0.370 | PE+PCB | 6 | 0.327 | PS | 6 | 0.311 |
|  |  |  |  |  |  | PS+PCB | 1 | 0.352 |
|  |  |  |  |  |  | PS+PCB | 2 | 0.359 |
|  |  |  |  |  |  | PS+PCB | 3 | 0.396 |
|  |  |  |  |  |  | PS+PCB | 4 | 0.346 |
|  |  |  |  |  |  | PS+PCB | 5 | 0.315 |
|  |  |  |  |  |  | PS+PCB | 6 | 0.361 |
|  |  |  |  |  |  | PS+PCB | 1 | 0.365 |
|  |  |  |  |  |  | PS+PCB | 2 | 0.328 |
|  |  |  |  |  |  | PS+PCB | 3 | 0.367 |
|  |  |  |  |  |  | PS+PCB | 4 | 0.366 |
|  |  |  |  |  |  | PS+PCB | 5 | 0.362 |
|  |  |  |  |  |  | PS+PCB | 6 | 0.398 |
|  |  |  |  |  |  | PS+PCB | 1 | 0.420 |
|  |  |  |  |  |  | PS+PCB | 2 | 0.310 |
|  |  |  |  |  |  | PS+PCB | 3 | 0.374 |
|  |  |  |  |  |  | PS+PCB | 4 | 0.424 |
|  |  |  |  |  |  | PS+PCB | 5 | 0.377 |
|  |  |  |  |  |  | PS+PCB | 6 | 0.366 |
