# Supplementary material for: Direct and indirect effects of different types of microplastics on freshwater prey (Corbicula fluminea) and their predator (Acipenser transmontanus)
Source: PLoS One. 2017 Nov 6;12(11):e0187664. doi: 10.1371/journal.pone.0187664 (PMC5673206; doi:10.1371/journal.pone.0187664)
Supplement: S1 Fig — A graph showing model predictions across polymers. (DOCX) [file pone.0187664.s010.docx]

*
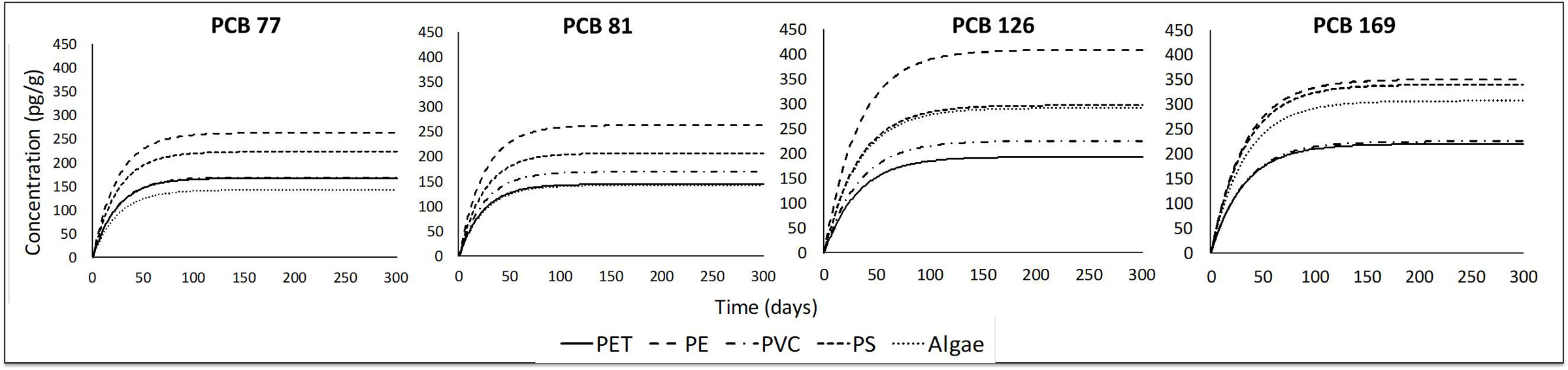
*

**S1 Fig. Model Predictions.** Predicted concentrations (pg/g; y axis) of PCB #81, 77, 126 and 169 (shown from left to right) in Asian clams fed algae, PET, PVC, PE (polyethylene) or PS (polystyrene) with PCBs over time (days; x axis) based on model predictions using COSMOtherm partition coefficients. Trends for PET are shown via a solid line, PE via a medium dashed line, PVC with a mixed dash/dotted line, PS with a small dashed line and algae with a dotted line.
